# Prostate Cancer Microvascular Routes: Exploration and Measurement Strategies

**DOI:** 10.3390/life13102034

**Published:** 2023-10-09

**Authors:** Fabio Grizzi, Mohamed A. A. A. Hegazi, Matteo Zanoni, Paolo Vota, Giovanni Toia, Maria Chiara Clementi, Cinzia Mazzieri, Maurizio Chiriva-Internati, Gianluigi Taverna

**Affiliations:** 1 Department of Immunology and Inflammation, IRCCS Humanitas Research Hospital, Rozzano, 20089 Milan, Italy; lunamohamed75@gmail.com; 2 Department of Biomedical Sciences, Humanitas University, Pieve Emanuele, 20072 Milan, Italy; gianluigi.taverna@humanitas.it; 3 Department of Urology, Humanitas Mater Domini, Castellanza, 21053 Varese, Italy; matteo.zanoni@materdomini.it (M.Z.); paolo.vota@materdomini.it (P.V.); giovanni.toia@materdomini.it (G.T.); mariachiara.clementi@mc.humanitas.it (M.C.C.); cinzia.mazzieri@materdomini.it (C.M.); 4 Departments of Gastroenterology, Hepatology & Nutrition, Division of Internal Medicine, The University of Texas MD Anderson Cancer Center, Houston, TX 77030, USA; mnchiriva@mdanderson.org

**Keywords:** prostate cancer, vasculature, microvessel density, biomarkers, fractals

## Abstract

Angiogenesis is acknowledged as a pivotal feature in the pathology of human cancer. Despite the absence of universally accepted markers for gauging the comprehensive angiogenic activity in prostate cancer (PCa) that could steer the formulation of focused anti-angiogenic treatments, the scrutiny of diverse facets of tumoral blood vessel development may furnish significant understanding of angiogenic processes. Malignant neoplasms, encompassing PCa, deploy a myriad of strategies to secure an adequate blood supply. These modalities range from sprouting angiogenesis and vasculogenesis to intussusceptive angiogenesis, vascular co-option, the formation of mosaic vessels, vasculogenic mimicry, the conversion of cancer stem-like cells into tumor endothelial cells, and vascular pruning. Here we provide a thorough review of these angiogenic mechanisms as they relate to PCa, discuss their prospective relevance for predictive and prognostic evaluations, and outline the prevailing obstacles in quantitatively evaluating neovascularization via histopathological examinations.

## 1. Introduction

Cancer research has undergone radical changes in recent years [1]. The challenge now is not the volume of foundational and clinical data at our disposal, but rather the management and interpretation of this surfeit of information. In spite of these advancements, prostate cancer (PCa) continues to pose a substantial global public health concern [2,3]. Cancers of the prostate, lung, and bronchus, as well as colorectal cancers, collectively constitute nearly half of all new male cancer cases, with PCa alone responsible for 27% of these diagnoses [4]. Critical to tumor expansion, advancement, and the metastatic spread of cancer cells is the availability of blood supply. New blood vessels are essential for delivering oxygen and nutrients to the expanding tumor mass and for eliminating cellular waste products [5,6]. Angiogenesis, defined as the formation of new blood vessels branching out from pre-existing vascular structures, is a complex dynamic process. It is a natural physiological phenomenon observed in scenarios such as fetal development, wound repair, and endometrial hyperplasia linked with the menstrual cycle [6,7,8]. Under physiological conditions, angiogenesis is tightly regulated, being activated for brief durations and subsequently completely suppressed. In certain non-malignant conditions, such as lobular capillary hemangioma or keloid formation, angiogenesis is a transient event [9]. Nevertheless, various human diseases exhibit a sustained upregulation of angiogenesis [10,11]. However, in the context of tumors, once initiated, angiogenesis persists indefatigably until either the entire tumor is eradicated or the host organism dies [7]. The regulation of angiogenesis is achieved through a finely calibrated equilibrium of pro- and anti-angiogenic factors [11]. These factors are secreted by an array of cells, including cancer cells, endothelial cells (ECs), and stromal cells (SCs) [12,13]. The relative impact of these factors is likely variable, dependent on the type and location of the tumor, as well as its stages of growth, regression, and recurrence. 

The concept of “Angiogenesis” can be traced back to the work of British surgeon John Hunter (1728–1793) [14]. In his book “A Treatise on the Blood, Inflammation, and Gun-shot Wounds”, released in 1794, Hunter outlined the formation of new blood vessels, effectively prefiguring the contemporary understanding of angiogenesis. He noted enhanced vascularization not merely during the developmental stages of young animals but also under pathological circumstances and in healing mechanisms [15,16]. Subsequently, in 1826, Dutch anatomist and physiologist Jacobus Ludovicus Conradus Schroeder van der Kolk (1797–1862) contributed further to this subject. In his report titled “Observationes Anatomico-Pathologici et Practici Argumenti”, Schroeder van der Kolk described the presence of novel and anomalous vascular structures in both tumors and parasitic diseases [17].

In a significant proportion of cancers, the vasculature exhibits abnormalities across various dimensions of structure and function [18]. These aberrant tumor vessels can limit the performance of immune cells within the tumor milieu and compromise the transport and distribution of chemotherapeutic agents and oxygen. Resulting conditions such as interstitial hypertension, hypoxia, and acidosis—each an outcome of distorted vascular architecture and function—create an ecosystem conducive to tumor advancement and metastatic spread [19]. Involved in these processes are key angiogenic modulators including, but not limited to, the vascular endothelial growth factor (VEGF) family and their receptors (VEGF-Rs), basic fibroblast growth factor (b-FGF), and the angiopoietin family. These factors contribute to the development of hypoxic and acidic regions within the tumor [20]. Contrary to the prevailing notion that the ECs composing the tumor vasculature are genetically stable, the nature of this vasculature appears to be far more unpredictable [21]. These specific conditions influence the production of pro- and anti-angiogenic molecules, diminish the efficacy of therapeutic interventions, and facilitate the selection of sub-populations of cancer cells that exhibit heightened aggressiveness and metastatic capabilities. The role of angiogenesis in PCa continues to be a subject of ongoing debate and investigation [13,22,23,24]. Angiogenesis has been identified as a critical factor in both the development and propagation of PCa [25]. PCa cells are capable of synthesizing an array of bioactive molecules such as matrix metalloproteinases (MMPs) [26], VEGF, transforming growth factor β (TGF-β), and cyclooxygenase-2 (COX-2). Given that overexpression of VEGF-A has been correlated with poor prognosis and metastatic potential in PCa, the majority of clinical trials focused on anti-angiogenic interventions in PCa have targeted VEGF-A specifically [27]. A randomized phase II clinical trial involving bevacizumab, which included 99 hormone-sensitive PCa patients, demonstrated enhanced relapse-free survival when bevacizumab was administered in conjunction with hormone deprivation therapy [25]. Conversely, a randomized, double-blind, placebo-controlled phase III study, encompassing 1050 patients suffering from PCa, indicated modest gains in progression-free survival but no significant enhancement in overall survival (OS) for metastatic, castration-resistant PCa when bevacizumab was used in combination with docetaxel chemotherapy and prednisone hormonal therapy [28]. These findings imply that bevacizumab may offer some clinical benefits, particularly in cases of hormone-sensitive recurrent PCa. However, in hormone-resistant, refractory tumors—where traditional therapeutic options often prove ineffective—incorporating bevacizumab into the treatment regimen does not appear to yield any discernible clinical advantages.

Despite the elevated expression of VEGF-A in advanced stages of PCa, therapeutic approaches aimed at inhibiting the VEGF-A pathway have not yielded substantial treatment advantages [29]. Unlike many other types of solid tumors, PCa has also demonstrated limited responsiveness to immune checkpoint inhibitors, including PD-L1 blockade. Recent research by Wang et al. revealed that blocking the interaction between VEGF and neuropilin-2 (NRP2) through antibody-mediated methods led to reduced PD-L1 expression in PCa cells and triggered antitumor immune responses in mouse models [27]. Several plausible rationales exist to explain the observed resistance to anti-angiogenic treatments in PCa [30,31]. One such reason is the redundancy present within angiogenic pathways. When one pathway is targeted for inhibition, compensatory mechanisms may activate alternative pathways, thereby diminishing the effectiveness of the intervention. Another contributing factor to treatment resistance is the molecular heterogeneity inherent to PCa. Presently, there is an absence of reliable biomarkers capable of identifying patients who are most likely to benefit from anti-angiogenic treatments, as well as those that could be utilized to evaluate therapeutic responsiveness. The genetic composition related to the VEGF-A pathway, as well as variations in VEGF-A or its associated receptors, are currently under consideration as potential indicators for predicting therapeutic outcomes; however, these possibilities have not yet been empirically validated. Moreover, elevated concentrations of VEGF-A have been identified in the urine of PCa patients, suggesting its possible utility as a prognostic marker for forecasting the course of hormone-refractory PCa and associated survival rates [32,33]. Ongoing and future phase III trials are anticipated to delineate specific patient subgroups that could derive benefit from anti-angiogenic interventions. In a recent study, Jaraini et al. have illuminated how divergent biological processes in various cell types contribute to angiogenesis. Their findings offer insights into the prospective utility of targeted anti-angiogenic strategies. Specifically, the authors underscore the significance of genes such as ACKR1, AQP1, and EGR1, which manifest strong patterns of cell type-dependent overexpression. These genes may offer valuable diagnostic and prognostic markers in the context of PCa [34]. Further comprehensive research has established the role of the chemokine axis CXCL12-CXCR4 as a central factor in bone metastasis specifically related to PCa [35]. This axis thus represents another potential target for diagnostic and therapeutic strategies aimed at managing this complex disease. The activation of the CXCL12-CXCR4 pathway has been shown to elevate markers of epithelial-to-mesenchymal transition (EMT), namely E-cadherin and Vimentin, in PCa cells. This elevation leads to a loss of cellular adhesion, coupled with the acquisition of invasive and migratory capabilities, thereby promoting the progression of PCa. Furthermore, CXCL12-CXCR4 serves as an indicator of bone metastasis in PCa and influences angiogenesis in the tumor via ECs. Recent findings suggest that CXCL12 could serve as a novel marker for ECs specific to PCa, as its expression was markedly elevated in tumor-associated ECs in comparison to in vitro ECs. In preclinical trials utilizing a mouse model of PCa xenografts, inhibition of the CXCL12-CXCR4 axis demonstrated anti-angiogenic effects, evidenced by a reduction in both the number and density of blood vessels [36]. Additionally, the suppression of CXCL12 expression led to a decrease in the levels of MMP9, which is mediated by zinc-finger transcription factors. This resulted in a subsequent reduction in the metastatic potential of PCa cells [37]. Thus, targeting this pathway could present a novel approach for mitigating both angiogenesis and metastasis in the context of PCa.

**Figure 1:**
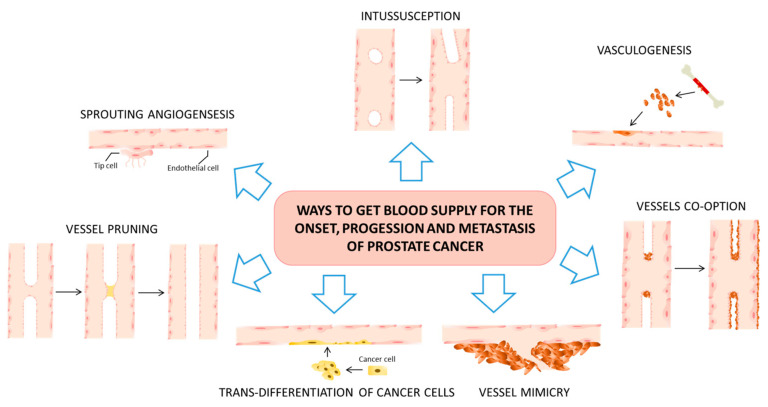
Tumor angiogenesis predominantly manifests through seven distinct mechanisms. Sprouting angiogenesis serves as the archetypical process in both physiological and pathological angiogenesis. In this mode, new vascular branches emerge from pre-existing blood vessels and ultimately infiltrate the tumor tissue via the migration of tip cells and the proliferation of stem cells. Notably, vessel co-option and vasculogenic mimicry are closely tied to tumor invasion, metastasis, and resistance to traditional anti-angiogenic therapies. Intussusceptive angiogenesis is characterized by the formation of a dual lumen that subsequently bifurcates into two separate vessels, which infiltrate the tumor tissue. Vasculogenesis, on the other hand, entails the recruitment of either bone marrow-derived or vessel wall-resident endothelial progenitor cells. These progenitor cells undergo differentiation into ECs, contributing to the formation of new vascular networks. Vasculogenic mimicry represents another unique avenue, wherein tumor cells extend to create a simulated vascular lumen. These simulated lumens then integrate into pre-existing blood vessels, thereby facilitating the transport of erythrocytes and oxygen into the tumor tissue. Lastly, there exists another process, known as the trans-differentiation of cancer stem-like cells. In this mechanism, these specialized cells acquire endothelial phenotypes and transform into endothelial-like cells via a process known as epithelial–endothelial transformation. Each of these angiogenic modes holds distinct implications for tumor growth, metastatic potential, and responsiveness to anti-angiogenic therapies. As such, understanding these varied processes is crucial for devising more effective treatment strategies and improving prognostic outcomes.

Various alternative mechanisms for tumor angiogenesis have been observed across different types of neoplasia, including PCa. These mechanisms encompass a wide range of processes, such as sprouting angiogenesis, vasculogenesis, intussusceptive angiogenesis, vascular co-option, mosaic vessels, vasculogenic mimicry, the transformation of cancer stem-like cells into tumor ECs, and vascular pruning [38,39,40]. In this context, we briefly review different ways through which tumors, specifically PCa, secure their blood supply (Figure 1). These mechanisms are critical for the initiation, progression, and metastatic spread of the disease. The mechanisms also hold potential predictive and prognostic implications. 

The primary method through which PCa secures its blood supply for growth is primarily facilitated by two processes: sprouting angiogenesis and vasculogenesis (the transformation of precursor cells into endothelial cells, thereby forming new vascular networks from scratch). It is worth noting that several aspects related to the blood supply in PCa have not been thoroughly explored; much of the existing research has either relied on in vitro studies or been conducted within the context of other physiological or pathological processes. The role of angiogenesis in metastatic castration-resistant prostate cancer (mCRPC) has garnered significant attention as a potentially actionable biological mechanism for drug development. However, attempts to target angiogenesis have not successfully extended OS rates among mCRPC patients, despite encouraging findings from both preclinical experiments and early-stage clinical trials. Additional research has revealed that PCa cells not only release a variety of angiogenic factors to stimulate the growth of blood vessels but also directly create tumor-associated vascular channels, a phenomenon termed as vasculogenic mimicry. Luo et al. [41] have documented that PCa cases exhibiting vasculogenic mimicry are more prone to developing bone metastases.

It is also crucial to note that there exist substantial challenges in the quantitative assessment of neovascularization through histopathological analysis. These challenges stem from the complex and multifaceted nature of tumor angiogenesis, which complicates the identification of reliable markers for assessing overall angiogenic activity in PCa. As a result, efforts are ongoing to delineate these processes more precisely and identify more accurate measures for both prediction and prognosis in the realm of PCa angiogenesis.

## 2. Prostate Cancer and the Sprouting Angiogenesis

Sprouting angiogenesis is frequently identified as a pivotal method for the vascularization of tumors, thus making it a principal focus for therapies aimed at inhibiting angiogenesis [39,42]. This mechanism involves a complex and regulated series of events beginning with the selection of a singular EC, commonly known as the “tip cell”, from existing vasculature. This selected tip cell then navigates its way out of a dormant state to form a new blood vessel [43]. The entire sequence takes an extended period, often exceeding 24 h, before the newly formed capillary loop can be perfused and subsequently integrated into the existing vascular network. During the occurrence of sprouting angiogenesis, the expansion of vessels is propelled by both the proliferation and migration of ECs that originate from pre-established vascular structures. The process adheres to a structured pathway that involves the migration of the endothelial tip cell toward an angiogenic stimulus, commonly growth factors, which are secreted by tumor cells and their surrounding stroma. Following migration, there is an increased rate of EC proliferation, contributing to the sprouting in tumors. Subsequently, a narrow, slit-like lumen forms that aligns with the lumen of the originating or “mother” vessel. The maturation phase of this new vessel involves the migration of proliferating pericytes from the “mother vessel”, which travel along the basement membrane of the new sprout [19]. When it comes to sprouting angiogenesis in the context of PCa, the scientific understanding remains limited. Existing research has shown that the binding of VEGF-A to its respective cell membrane receptors on endothelial tip cells plays a crucial role in guiding vessel sprouting. This is achieved by facilitating the localized degradation of the extracellular matrix (ECM), thereby allowing cells to invade this matrix. Moreover, VEGF-A gradients serve to stimulate the mobility of tip cells. Additional molecules, such as delta ligand-like 4 (DLL4), act to inhibit the sprouting of subsequent stalk cells, while concurrently guiding the deposition and remodeling of the ECM [44,45]. Regarding therapeutic strategies aimed at advanced PCa, the approach of selectively targeting immature and newly sprouting vessels has been suggested. This is achieved by inhibiting the synthesis of angiogenic factors and interfering with the integrity of the ECM [46,47,48,49]. Complementary approaches such as hormonal ablation, intermittent androgen suppression, chemotherapy, and radiation therapy—which have the effect of damaging nascent vascular structures and disrupting tumor–stroma interactions—are also under consideration for enhancing the effectiveness of experimental anti-angiogenic agents [46,47,48].

## 3. Prostate Cancer and the Intussusceptive Angiogenesis

Intussusceptive microvascular growth (IMG), alternatively termed intussusceptive angiogenesis or vessel splitting, represents another principal mechanism of angiogenesis. This process is characterized by the creation of new vessels through vascular invagination, the formation of intra-luminal pillars, and subsequent vessel splitting [50,51]. Unlike sprouting angiogenesis, IMG has been demonstrated to be both more rapid and more economical, and it does not principally rely on EC proliferation, degradation of the basement membrane, or invasion of the ECM [38,52]. One key distinction between IMG and sprouting angiogenesis is that IMG can only operate within existing vascular networks. The most salient attribute of IMG appears to be its capacity to amplify the intricacy and density of the pre-existing tumor microvascular network, which has been primarily constructed through sprouting angiogenesis. Moreover, IMG has the ability to expand the available endothelial surface area, thus facilitating further sprouting. Initially, vascular intussusception was primarily associated with physiological vascular development [53], but recent studies have extended its implications to experimental tumor models. It has been posited that the mechanism of angiogenesis may transition from sprouting to intussusception as a strategy for rapid vascular expansion [54]. Intussusception has been implicated in three processes of vascular growth and remodeling: (a) IMG allows for the accelerated enlargement of the capillary plexus, offering an extensive endothelial surface for metabolic exchanges; (b) intussusceptive arborization is a process that leads to alterations in the dimensions, positioning, and morphology of preferentially perfused capillary segments, thereby forming a hierarchical vascular tree; (c) intussusceptive branching remodeling contributes to the optimization of the geometrical design of the supplying vessels, enhancing the pre- and post-capillary flow characteristics. Currently, the molecular mechanisms driving IMG remain insufficiently elucidated. Likewise, the occurrence and role of IMG in the context of PCa have yet to be thoroughly investigated. It is understood that local factors, such as intravascular shear stress, have the potential to trigger a sequence of either physiological or pathological responses in ECs. One such response could be the formation of new capillaries through the development of tissue pillars [55]. Additionally, intussusceptive angiogenesis appears to be orchestrated by a range of cytokines, including but not limited to Platelet-Derived Growth Factor-BB (PDGF-BB), angiopoietins and their corresponding Tie receptors, TGF-β, Monocyte Chemotactic Protein-1 (MCP-1), and ephrins along with Eph-B receptors. In a comparative study by Díaz-Flores et al., the behavior of pericytes and CD34^+^ SCs/telocytes (TCs) was assessed in the context of two distinct processes: sprouting angiogenesis and intussusceptive angiogenesis [56]. In these two mechanisms, the modalities of neo-vessel formation diverge substantially. In sprouting angiogenesis, neo-vessels are generated by vessel sprouts extending outward, whereas in intussusceptive angiogenesis, they form through the development of intravascular or transluminal tissue pillars that subsequently divide and restructure existing vessels. In the realm of sprouting angiogenesis, proliferative perivascular CD34^+^ SCs/TCs serve as a vital source of SCs during reparative granulation tissue formation as well as a source for cancer-associated fibroblasts (CAFs) within tumors. Conversely, these CD34^+^ SCs/TCs play a less significant role as precursor cells in the context of intussusceptive angiogenesis. Malfunction of these angiogenic mechanisms is implicated in various pathological conditions, including neoplasms, thereby having therapeutic relevance. Notably, it has been reported that intussusceptive angiogenesis is resistant to anti-VEGF strategies [57]. Initially identified in pulmonary tissue, intussusceptive angiogenesis has also been observed in multiple types of tumors such as melanoma, colorectal cancer, glioma, and mammary tumors. However, the molecular mechanisms governing this angiogenic process within the vascular system remain largely undefined. Additionally, the incidence and potential clinical relevance of intussusceptive angiogenesis in the context of PCa are yet to be comprehensively investigated [58,59,60].

## 4. Prostate Cancer and the Vasculogenesis

The concept of “vasculogenesis” refers to a principal mechanism governing the de novo formation of blood vessels, characterized by the development of a capillary-like network originating from either dispersed or monolayered populations of ECs [61,62]. Traditionally, vasculogenesis has been considered a phenomenon restricted to the early stages of vascular development. However, recent research indicates that bone marrow-derived endothelial progenitor cells are capable of homing to areas of both physiological and pathological neovascularization and differentiating into mature ECs [63]. These endothelial progenitor cells are often mobilized from the bone marrow by cytokines produced by tumor tissues [19]. VEGF stands as the most extensively characterized among these cytokines, playing a critical role in mediating vasculogenesis by stimulating EC growth, migration, and mitosis. It also has significant implications in the pathogenesis, progression, and metastasis of cancers. Wang et al. have recently assessed the prognostic utility of VEGF in the context of PCa [64]. In progressing tumors, elevated levels of circulating VEGF were observed, and these levels showed a correlation with the number of endothelial progenitor cells present in the bloodstream. Additionally, Yang et al. reported that the human bone metastatic LNCaP-derivative C4-2B PCa cell line exhibited a higher level of VEGF expression compared to its parental primary PCa cell line, LNCaP [65]. Bone metastasis constitutes a major cause of cancer-related mortality in cases of advanced PCa. Several studies have revealed that PCa cells appear to manipulate their microenvironment in the early hypoxic stages to facilitate the migration of bone-marrow-derived endothelial progenitor cells (BM-EPCs) and vasculogenesis through cytokine secretion [66,67]. Experimental evidence also suggests that Acidic Medium (AM) stimulates the secretion of VEGF from PC-3 cells, and AM-conditioned medium (CMAM) enhances BM-EPC vasculogenesis by elevating cell viability, migration, and tube formation. This promotion is achieved through the activation of phosphorylation pathways involving VEGFR-2, Akt, and P38 when the pH of Normal Medium-conditioned medium (CMNM) is similarly modulated to that of CMAM [68]. In the therapeutic context, tumor irradiation disrupts local angiogenic processes, prompting the tumor to shift towards vasculogenesis for post-irradiation regrowth. The irradiation procedure induces a significant influx of CD11b+ myeloid cells, predominantly macrophages, into the tumor microenvironment (TME). These macrophages are instrumental in the genesis of new blood vessels following irradiation, thereby contributing to tumor recurrence. Chen et al. documented that radiotherapy diminishes vascular density and induces hypoxia, leading to the subsequent aggregation of macrophages in TRAMP-C1 PCa [69].

## 5. Prostate Cancer and the Vessel Co-Option

In the process of sprouting angiogenesis, the growth of vessels is facilitated by the proliferation and migration of ECs from already existing blood vessels. Contrarily, vessel co-option does not involve the formation of new blood vessels; rather, cancer cells appropriate existing vasculature for their own growth and subsequent invasion into healthy tissue [40,42]. The concept of vessel co-option was originally introduced by Francesco Pezzella in a seminal study published in the mid-1990s, where he elucidated an “alveolar or putative nonangiogenic” pattern of vascularization [70]. The existence of “mosaic” vessels—comprising both ECs and tumor cells as constituents of the luminal surface—has significant ramifications for aspects like metastasis, drug delivery, and anti-vascular therapeutic approaches [71].

Vessel co-option serves as an alternative method for tumors to secure a blood supply [19]. This mechanism, which involves the utilization of already existing blood vessels, was initially observed in the brain, one of the most richly vascularized organs in the human body. This type of vascularization has been implicated in aiding the infiltration of human gliomas [72]. Although Thompson proposed as early as 1987 that tumors might assimilate the vasculature of host tissue capillaries [73], it was not until a 1999 study by Holash et al. that the concept of vessel co-option was formally introduced [74,75]. In this mechanism, the tumor cells encircle and commandeer existing vessels, without initiating new sprout formation or immediate angiogenesis to bolster the tumor [19]. This strategy of co-opting pre-existing vessels may be sustained throughout the entire lifespan of both primary and metastatic tumors. For instance, in cutaneous melanoma, tumors do not demonstrate signs of directed angiogenesis but rather seem to grow by co-opting the extensive vascular plexus in the surrounding connective tissue [76]. The subject of vessel co-option’s role in tumorigenesis remains a point of academic debate. A study by Lugassy et al. found that specific cancer cell lines, including PC-3 human PCa cells and B16F10 murine melanoma cells, displayed a unique angiotropic pattern of spreading along the external surfaces of blood vessels [77]. This revealed the existence of “mosaic” vessels, in which both ECs and tumor cells form the inner lining, posing implications for metastasis, drug delivery, and antivascular treatments [71]. These mosaic vessels are defined by the presence of GFP-expressing tumor cells in apparent direct contact with the vascular lumen, as demonstrated by the lack of CD31/CD105 immunoreactivity on the vessel surface. Such vessels exhibit a disruption in the normally stained endothelial layer, with GFP-expressing tumor cells occupying the non-stained regions. Multiple studies across different types of cancer—including melanoma, breast, ovarian, lung, prostate, and glioblastoma—have described the formation of perfusable, vasculogenic-like networks made entirely of tumor cells, a phenomenon termed “vascular mimicry” (VM). In these settings, the lack of an endothelial layer grants tumor cells the opportunity to enter the circulatory system without requiring trans-endothelial migration.

## 6. Prostate Cancer and the Vasculogenic Mimicry

Originally identified in the context of uveal melanoma, VM refers to the capacity of aggressive tumor cells to undergo trans-differentiation. This allows them to adopt EC behaviors, facilitating the creation of vascular networks and a microcirculation system that is autonomous from non-cancerous host cells [78]. VM is generally assessed in clinical samples through immunohistochemical (IHC) analysis focusing on Periodic Acid Schiff (PAS)-positive CD34^−^ vessels. Here, PAS serves to stain basement membranes, which include components like laminin, collagen, and glycogen, while CD34 functions as a marker specifically for ECs. A comprehensive systematic review that incorporated meta-analysis and covered 3062 patients across multiple types of cancer demonstrated that the presence of VM correlates with a poorer 5-year OS rate. Furthermore, VM is associated with the facilitation of more aggressive forms of the disease and an increased propensity for metastatic spread [78]. Thus, the clinical implications of VM are significant, as it serves as an indicator of both disease severity and the likelihood of unfavorable outcomes, including metastasis.

Aggressive neoplasms can self-sustain by generating their own vascular-like structures for blood and nutrient supply. Maniotis et al. articulated in 1999 that highly invasive melanoma cells have the capacity to transform into multiple cellular forms, including those with endothelial-like properties that enable them to construct vessel-like conduits for blood supply [79]. These conduits are developed de novo and do not constitute genuine blood vessels, as noted in multiple studies [19,79,80]. The concept of VM is used to define this phenomenon, wherein highly invasive and genetically unstable tumor cells generate these channels without involving ECs. In its original description in melanoma, these channels are enveloped by a thin basal lamina akin to the wall of an actual vessel, albeit lacking ECs [19]. VM can manifest in two distinct forms: the tubular type, which can be mistaken for genuine endothelial-lined blood vessels, and the patterned matrix type, which bears no morphological or topological resemblance to actual blood vessels. In both instances, tumor cells line the external surfaces of these channels [81,82]. Despite the flow of blood plasma and red blood cells in these channels, neither inflammatory cells nor necrosis are present [82]. VM is observed across a diverse range of tumors, including but not limited to breast cancer, hepatocellular carcinoma, osteosarcoma, melanoma, ovarian carcinoma, and PCa [65,80,83]. In 2002, Sharma et al. provided corroborative data that VM is evident in heterogeneously invasive PCa cell lines and in aggressive tumors in both rats and humans [84]. Through GFP tagging of prostatic cellular subtypes, the study disclosed unique interplays between epithelial- and fibroblast-like tumor cells in the construction of perfusable vasculogenic-like networks. Moreover, channels lined by PCa cells were also observed in vivo in high-grade tumors, sometimes situated in close proximity to traditional endothelial-lined vasculature [84]. Liu et al. examined VM’s significance in the advancement of PCa and ascertained that VM predominantly occurs in high-risk PCa patients, serving as an independent prognostic marker for unfavorable outcomes [83]. A recent in vitro study suggested that VM in PCa could play a significant role in bone metastasis [85]. Multiple reports have indicated that VM is intrinsically linked to the progression and metastasis of cancer and is associated with poor prognostic factors in cancer patients. A study by Han et al. has even investigated the potential inhibitory role of resveratrol on VM in human PCa PC-3 cells, proposing that resveratrol might suppress VM by inhibiting the EphA2/twist-VE-cadherin/AKT signaling cascade [86].

VM has been strongly linked with the invasive and metastatic characteristics of PCa. There is a significant correlation between VM formation and various prognostic markers in high-risk PCa patients, such as the Gleason score, lymph node metastasis, and distant metastasis [83,87,88]. Notably, the survival rates, both in terms of OS and disease-free survival, are notably diminished in patients exhibiting VM-positive PCa compared to those with VM-negative PCa. These findings collectively underscore the prognostic relevance of VM, suggesting that its presence is an indicator of adverse outcomes in cancer patients. Given these associations, targeting VM could serve as a strategic approach to circumvent the limitations of existing anti-angiogenic treatments. The co-administration of anti-VM therapies with traditional anti-angiogenic treatments might yield synergistic anti-cancer effects. Ideally, drugs that can concurrently target both VM and angiogenesis would offer the most comprehensive therapeutic approach. In this context, a study by Han and Lee has shown the role of Specificity Protein 1 (Sp1), a transcription factor implicated in various aspects of tumor progression. According to their research, Sp1 influences VM formation by interacting with the twist/VE-cadherin/AKT signaling pathway in human PCa cells [89]. This finding provides further avenues for exploring targeted therapies that could disrupt both VM and angiogenesis, offering a more holistic treatment paradigm for advanced PCa.

## 7. Prostate Cancer and the Trans-Differentiation of Cancer Stem-like Cells into Tumor Endothelial Cells

Recent research has highlighted the role of Cancer Stem Cells (CSCs) and Epithelium-to-Endothelium Transition (EET) in the process of tumor angiogenesis [7]. EET, a specialized form of EMT, has been shown to facilitate VM formation by enhancing tumor cell plasticity, restructuring the ECM, and establishing connections between VM channels and the host’s blood vessels. Although initially understood as crucial in heart development, it has become evident that EET can also transpire postnatally in a variety of pathological scenarios, such as cancer and cardiac fibrosis [90]. Within the TME, EET serves as a substantial contributor to the generation of CAFs, constituting up to 40% of the CAF population [90]. CAFs play a multifaceted role in advancing tumor progression. They not only modify the TME by depositing diverse ECM molecules but also secrete paracrine factors that directly influence the behavior of various cellular constituents within the tumor. Moreover, CAFs emit potentially oncogenic signals like TGF-β and are a key source of host-derived VEGF, which stimulates angiogenesis [91]. In the context of PCa, CAFs have been demonstrated to be pivotal in the disease dynamics [92]. Both in vitro and in vivo studies have shown that CAFs support the progression of low-tumorigenic prostate adenocarcinoma cells, driving them toward castration resistance and ultimately leading to bone metastasis [93,94]. It is noteworthy that PCa has a predilection for forming bone metastasis, in contrast to neuroendocrine PCa, which can also metastasize to soft tissues like the lungs or liver [95,96]. The transition of PCa to a metastatic and therapeutically resistant state involves intricate interactions between cancer cells, CAFs, and the neo-formation of blood vessels [97]. Although the precise pathogenesis and underlying mechanisms of endothelial-to-mesenchymal transition in PCa remain elusive, Chang and Song have provided evidence that Plasmacytoma Variant Translocation 1 (PVT1), a newly identified long non-coding RNA, plays an instrumental role in promoting PCa invasion and metastasis by modulating endothelial–mesenchymal transition [98]. Therefore, understanding these complex interactions may offer novel therapeutic targets for the effective management of advanced PCa.

## 8. Prostate Cancer and Pruning

Pruning constitutes a fundamental process occurring at all levels of the vascular network, serving as a remodeling mechanism aimed at reducing the energetic demands associated with tissue perfusion [99,100]. Unlike sprouting, which emerges as a cellular response to localized needs, pruning is thought to have evolved as a strategy to minimize the energy required for blood flow. This optimization is achieved by restructuring the existing vascular network to reduce its overall length and surface area, yet without compromising its essential functionality [99]. While the criteria for selecting specific vessel segments for pruning remain ambiguous, the general sequence in developmental remodeling involves ECs constricting the vessel. This constriction results in diminished and eventually static blood flow, leading to vessel occlusion. The ECs affected by this stagnation undergo a combination of apoptosis and migration, causing the occlusion to rupture and thereby form two distinct vessel segments [101]. 

In PCa, particularly with PC3/2G7 tumors, extended treatment using axitinib has revealed an apparent evasion from anti-angiogenic effects. This escape manifests as a reinitiation of neovascularization and, consequently, tumor growth resumption. Multiple mechanisms have been proposed for such evasive behavior from VEGFR-targeted anti-angiogenic treatments. One significant mechanism involves the tumor’s capacity to upregulate alternative proangiogenic molecules, thereby rendering angiogenesis VEGF-independent. This adaptation may be partially triggered by the hypoxic conditions induced by vessel pruning, which in turn activate factors promoting tumor vascularization [102]. The hypoxia induced by therapeutic interventions also critically influences the selection of tumor cells that can withstand, or even flourish, in low-oxygen conditions [103]. Further studies have identified that pruning mechanisms in PCa can actually enhance the efficiency of the existing vasculature during radiation treatment. Research conducted by Potiron et al. demonstrated that radiation-induced vascular pruning in PCa led to a simplification of the pre-existing vascular network while improving tissue perfusion [104]. Contrary to expectations, their findings did not identify specific hallmark features associated with normalized blood vessels, such as branching, microvessel density (MVD), tortuosity, or variations in vessel diameter [105]. Hence, these observations imply a nuanced understanding of pruning mechanisms and their implications, particularly in therapeutic contexts for PCa.

## 9. The Inhibition of Angiogenesis in Prostate Cancer

According to the specific target addressed by the therapeutic agent, the approach to inhibiting angiogenesis can be categorized into two principal classifications: direct and indirect inhibition [106]. Direct inhibitors focus on affecting the proliferating endothelial cells, whereas indirect inhibitors aim to modulate either the tumor cells themselves or the associated stromal cells within the tumor environment.

Small molecular fragments (i.e., arrestin, tumstatin, canstatin, endostatin, and angiostatin) function as direct inhibitors by curtailing EC proliferation and migration. These processes are typically induced by factors such as Vascular Endothelial Growth Factor-A (VEGF-A), basic Fibroblast Growth Factor (bFGF), Platelet-Derived Growth Factor (PDGF), and interleukins [107]. Conversely, indirect angiogenesis inhibition operates through a complex interaction between either the tumor cells or the stromal cells and the angiogenic process itself. An example of this is the inhibition of the Epidermal Growth Factor Receptor (EGFR), a tyrosine kinase receptor. When expressed and activated in tumor cells, EGFR stimulates the production of interleukins, which have been shown to encourage angiogenesis within the tumor. Therefore, obstructing the expression and/or activity of EGFR serves as a strategy for the indirect inhibition of angiogenesis [108]. 

VEGF-A has been shown to be markedly overexpressed in PCa and is correlated with unfavorable outcomes and metastatic progression. As a result, the majority of clinical studies focusing on anti-angiogenic therapies in PCa have targeted VEGF-A. Notable among these therapies are Bevacizumab, a recombinant humanized monoclonal antibody designed to neutralize VEGF-A; Aflibercept, which sequesters circulating VEGF-A; and Lenalidomide, which operates through a multi-faceted mechanism that includes the inhibition of VEGF-induced PI3K-Akt pathway signaling.

From both histopathological and preclinical viewpoints, substantial evidence exists to support the pivotal role of angiogenesis in the onset and advancement of PCa. For instance, the presence of VEGFR2 has been identified as a marker indicative of PCa cases that are at elevated risk for progression [109,110]. Moreover, specific microRNAs associated with angiogenesis, such as let-7, miR-195, and miR-205 [111], are found to be deregulated and hold significant roles in the pathology of PCa. These microRNAs contribute to the complex regulatory landscape of angiogenesis, thereby affecting the development and progression of the disease.

Nevertheless, the heterogeneity of blood vessels within tumors has been identified as a significant contributing factor to the limited effectiveness of anti-angiogenic treatments. The results of such therapies to date have been moderately successful at best, providing only a temporary decrease in tumor growth before resistance develops. Furthermore, these treatments have typically resulted in marginal improvements in OS rates, despite a broad spectrum of medications having received approval from the U.S. Food and Drug Administration (FDA). The constrained efficacy of these anti-angiogenic strategies can be attributed to a variety of factors. Among these are the employment of alternative angiogenic pathways by the tumors themselves, as well as the onset of mechanisms that lead to treatment resistance.

Currently, medical professionals are actively investigating which subgroups of PCa patients are most responsive to angiogenesis inhibition therapies. Additionally, there is a concerted effort to bridge the division between experimental research (“bench”) and clinical application (“bedside”). Clinicians and researchers are exploring the synergistic effects of combining angiogenesis inhibitors with other well-established anti-cancer compounds. Several anti-angiogenic medications have already received regulatory approval and are presently employed in the treatment of various cancers. This situation raises a couple of crucial questions. Firstly, is it feasible to target the various mechanisms by which PCa tumors secure their blood supply through a single, unified therapeutic approach, or will alternative strategies need to be devised to achieve effective treatment? Secondly, there is the unresolved issue of whether anti-angiogenic treatments could prove efficacious in cases of refractory castration-resistant PCa, a subtype for which existing treatment options are notably limited.

## 10. The Measure of Neovascularity in Prostate Cancer Tissue

The proposition that angiogenesis is a prerequisite for tumor growth was initially postulated in 1971 by Moses Judah Folkman (1933–2008). He articulated this concept by stating, “Once a tumor “take” has been established, every subsequent expansion in the tumor cell population must be preceded by an increase in new capillaries that converge towards the tumor” [112,113]. This seminal idea laid the foundation for subsequent research into the intricate relationship between angiogenesis and oncogenesis.

No definitive biomarkers exist that can quantify the net angiogenic activity within the tumor. This absence of specific markers imposes limitations on the formulation of targeted anti-angiogenic treatment strategies. Nevertheless, it is plausible to conjecture that a thorough quantification of various facets of the tumor’s vascular architecture might offer valuable insights into its angiogenic potential. Therefore, while we are yet to identify precise indicators of angiogenic activity in PCa, the measurement and assessment of tumor vasculature may serve as a provisional approach to gauging angiogenic processes.

### 10.1. The Micro-Vessel Density Evaluation

A frequently measured variable in the vascular system of PCa is MVD. The method for quantifying MVD within tumors was originally introduced by Weidner in 1991 [114]. This metric serves as a valuable tool for the histological assessment of tumor angiogenesis. While it is primarily applied to surgical specimens, it is also used in diagnostic biopsy samples, which are the initial means for diagnosis but are obtained through invasive procedures. It should be noted that men who undergo prostate biopsies commonly face a range of complications, including but not limited to hematospermia, hematuria, and infection [115]. Over the past ten years, research has posited that MVD serves as a useful prognostic indicator in the context of PCa. It is also conjectured to provide insights into the extent of angiogenic activity within PCa tumors. Employing MVD scoring is recognized as a valuable, straightforward, and practical histological technique for the routine evaluation of PCa. MVD has been identified as a reliable forecaster of Biochemical Recurrence (BCR) in PCa patients. Particularly, cases of PCa in the earlier T stages, when marked by a higher MVD, show a correlation with BCR [116]. Furthermore, it is established that androgens significantly influence the regulation of MVD in the prostates of Sprague-Dawley rats. Specifically, a reduction in Dihydrotestosterone (DHT) levels is linked to a corresponding decrease in prostatic MVD. Conversely, an elevation in DHT levels tends to increase prostatic MVD [117]. In a similar vein, an incremental rise in estrogen levels has also been observed to progressively augment prostatic MVD.

**Figure 2:**
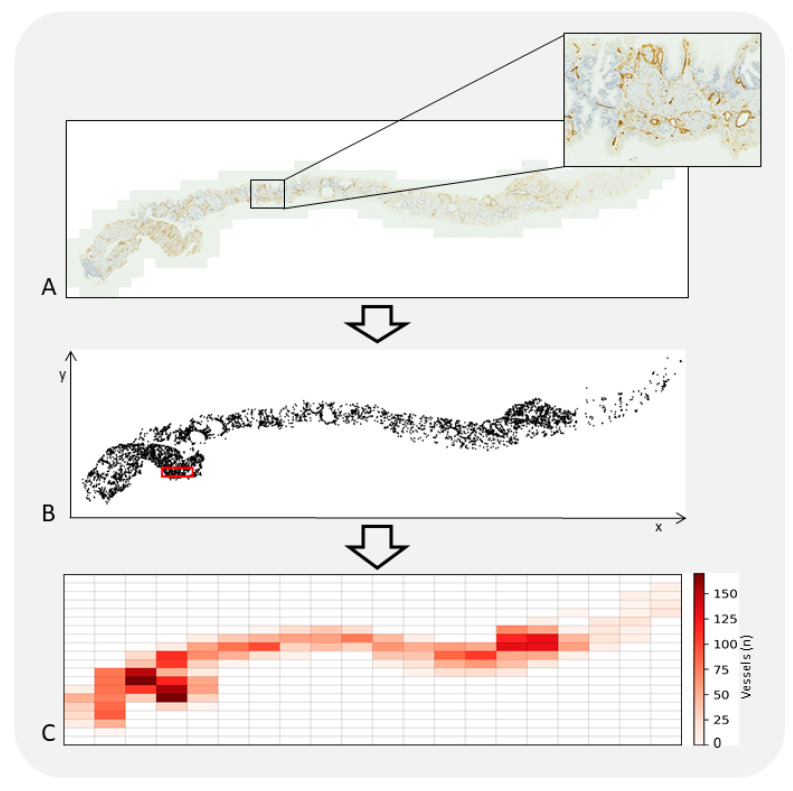
A tissue section of PCa was digitized employing a Zeiss Axioscan.Z1 microscope with a 20× objective lens (**A**). Vascular structures within the tissue were identified through the use of monoclonal antibodies targeting CD34. A computer-assisted image analysis system was utilized to ascertain the x-y coordinates of each identified vessel, thereby generating a two-dimensional spatial map of their distribution. The system additionally identifies a specific sub-region, delineated by a red outline, where the highest density of vessels is found; this area is the “hot-spot” (**B**). Within the tissue’s microenvironment, vessels are not uniformly distributed. This leads to a phenomenon known as vascular spatial heterogeneity, where areas rich in vessels are in proximity to areas with fewer or no vessels at all (**C**). This distribution pattern is influenced by a complex interplay of multiple variables, which are not only interconnected but can also vary over time and space. The irregular configuration of the vascular network poses a significant impact on microscopic examinations. It contributes to three forms of variability: intra-sample, inter-sample, and inter-observer. These variabilities are manifested during the qualitative assessment of tissue slides that have been stained for observation.

Nonetheless, MVD is not without its drawbacks. The inconsistency in results related to PCa is plausibly attributed to variations in study methodologies, encompassing factors such as the size of the patient cohort, the characteristics of the tumor, the methodology for selecting tumor areas for examination, the endothelial markers chosen, and the techniques employed for counting vessels [13,22,118,119,120]. Assessment of tumor areas for MVD can be approached in two distinct manners: (a) focused examination of specific microscopic “hot spots” characterized by maximal vascular density (Figure 2), and (b) a broader selection of randomized representative areas within the tumor. The former method is more commonly utilized due to its straightforwardness, although a consensus is lacking regarding optimal microscope magnification, the number of vascular “hot spots”, and threshold values to distinguish between low and high MVD. The latter approach, involving a wider scope of representative areas or even the entire tissue, may offer a more objective evaluation but necessitates a more laborious scrutiny (Figure 2). Empirical studies have shown that sustained exercise can influence tumor vascularization and decrease hypoxia in preclinical settings. This effect is possibly due to enhanced blood circulation during physical activity, as initially posited by McCollough et al. in an orthotopic PCa mouse model [121]. An investigation by Djurhuus et al. focused on the impact of a single episode of high-intensity interval training in patients with localized PCa who were scheduled for radical prostatectomy [122]. However, the authors did not observe any significant differences in tumor hypoxia or Natural Killer (NK) cell infiltration between groups [122]. Furthermore, they detected no notable correlation between MVD levels and either tumor hypoxia or NK cell infiltration. The conclusion drawn was that a solitary session of exercise is likely insufficient to affect tumor hypoxia or NK cell activity [122]. In a more constrained study on a limited sample of PCa tissues, Das and Mendonca examined both Mast Cell (MC) density and MVD [123]. Their findings indicated that the density of perilesional MCs and vascularity increased concomitantly with the severity of adenocarcinoma. This suggests that MCs may have a role in shaping the TME, influencing factors such as vascular density and tumor aggressiveness [123]. 

Despite its acknowledged role as a predictive indicator in untreated neoplasms, MVD has not been substantiated as a reliable metric for either guiding or assessing anti-angiogenic interventions [124]. Moreover, MVD fails to predict tumor behavior when subjected to anti-angiogenic therapies and is thus not an effective criterion for patient segmentation in clinical studies [124,125,126,127,128]. The fluctuations in tumor MVD do not necessarily align with variations in tissue or circulating levels of any specific pro-angiogenic factors. Interestingly, the MVD in tumor tissue is not necessarily elevated compared to its analogous normal tissue, which is not undergoing net growth. The effectiveness of anti-angiogenic agents cannot be straightforwardly inferred from changes in MVD during the course of treatment [124,129]. With regard to other metrics, commonly scrutinized Euclidean indices encompass parameters such as vessel area, wall area, lumen area, mean wall thickness-boundary, mean wall thickness-rosette, mean diameter-rosette, mean wall thickness-skeleton, and external diameter-skeleton. Research by Mucci et al. revealed only a tenuous relationship between MVD and the irregularity of the vascular lumen [130]. Conversely, a robust correlation was observed between vessel area and diameter [130]. Additionally, their study uncovered a pronounced inverse correlation between vessel dimensions and form. Specifically, tumors characterized by smaller vessel diameters and areas tended to exhibit more irregular vascular lumens, a finding which could be anticipated given the methodology employed for calculating vascular irregularity [130]. Nevertheless, it is important to note that these aforementioned metrics, including MVD, are constrained by the intricate biology defining tumor vasculature [131]. Moreover, the irregular geometrical structure assumed by the vascular network in tangible space defies measurement through Euclidean geometric principles, which are equipped to assess only regular and smooth objects that are scarcely encountered in natural settings [132].

Currently, there are no explicit biomarkers for evaluating angiogenic activity in PCa. However, it is plausible to postulate that vascular density serves as an indirect measure of intra-tumoral angiogenic processes. MVD is generally accepted as a viable proxy for angiogenic activity and has been validated as a prognostic determinant in a diverse array of malignancies, encompassing breast and colon cancers as well as malignant melanomas [81]. Initial studies have indicated that elevated MVD levels are correlated with advanced tumor grade and stage and are predictive of poorer outcomes in the context of PCa [83,84]. Additionally, ultrasonographic investigations focusing on hemodynamic indices have unveiled greater peak intensity in high-grade neoplasms [85]. Subsequent research, however, has not corroborated MVD as an autonomous prognostic factor in untreated tumors. Furthermore, no relationship has been definitively established between MVD and the efficacy of anti-angiogenic treatments specific to PCa [71]. Several plausible explanations for these discordant findings have been proposed. These include variations in the methodologies employed for counting micro-vessels, inconsistencies in the types of antibodies utilized, disparities in the sizes of the populations studied, and divergent levels of expertise and pathological understanding among researchers [99]. 

### 10.2. The Fractal Dimension Estimate of Prostate Cancer Vasculature

**Figure 3:**
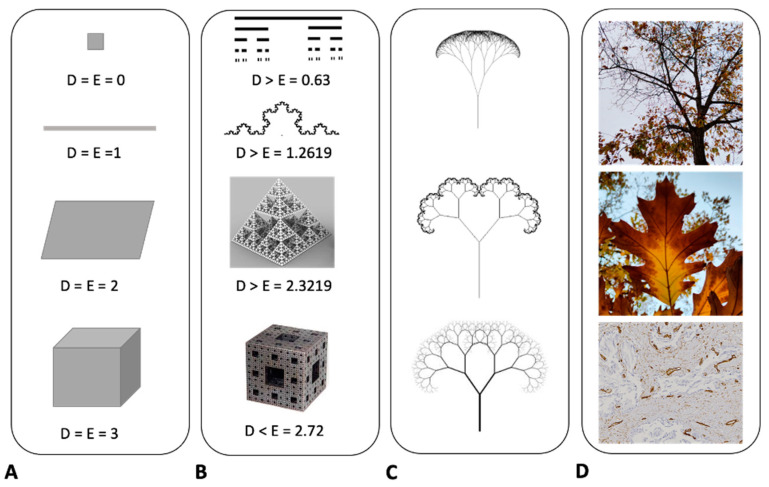
In Euclidean geometry, the dimensionality of objects is classified as follows. A point has a dimension of 0, a line has a dimension of 1, a surface is two-dimensional, and a solid object is three-dimensional (**A**). On the other hand, self-similar fractal objects exhibit non-integer dimensions. For example, the Cantor set has a dimension of 0.63, the Helge von Koch curve features a dimension of 1.2619, the fractal pyramid has a dimension of 2.3219, and the Menger Sponge possesses a dimension of 2.73 (**B**). Fractal mathematical trees serve as another illustrative example (**C**). These structures are generated through the continual iteration of a specific equation, resulting in a tree-like figure that exhibits similar characteristics across varying spatial scales. This notion of self-similarity extends to natural phenomena as well, such as the tree ramification, the vascular system in leaves, or two-dimensional vascularity in histological sections (**D**). These natural systems share key features with fractal objects; they exhibit irregular shapes, possess statistical self-similarity, have non-integer dimensions, and demonstrate the property of scaling. The attribute of scaling implies that the properties of these systems are scale-dependent; the characteristics manifest differently depending on the scale at which they are measured or observed. This underscores the complexity and adaptability of both mathematical and natural fractal systems.

Quantitative metrics elucidating the geometrical intricacy of various structures can indeed be extracted using fractal geometry, a mathematical framework initially put forth by Benoit Mandelbrot in 1975 [133,134,135,136]. Natural and anatomical fractals predominantly exhibit four distinguishing characteristics: (a) irregular contours, (b) structural self-similarity, (c) non-integer or fractal dimension, and (d) scaling behavior, implying that the properties observed vary depending on the scale at which measurements are taken (Figure 3). Two types of self-similarity can be identified: geometrical and statistical. An object is deemed geometrically self-similar when each of its smaller subdivisions is an exact replica of the entire object. Notable instances of geometrically self-similar constructs include the “snowflake” and the “curve”, which were originally described by Niels Fabian Helge von Koch, a Swedish mathematician, in 1904 [13,135]. Another example is the “Sierpinski triangle”, first outlined in 1915 by Polish mathematician Waclaw Sierpinski (1882–1969). On the other hand, statistical self-similarity pertains to biological and natural structures, where the constituent elements are not exact duplicates but exhibit the same kind of self-similarity. Various anatomical frameworks, such as the circulatory system, the biliary tree in the liver, neuronal dendritic structures, glandular ductal systems, cell membranes, and extracellular matrices in chronic liver diseases, have been illustrated as statistically self-similar entities [135]. Regarding the notion of “dimension”, it serves as a unique attribute of the object in question. Two distinct definitions of dimensionality have been posited. The first, termed “topological dimension”, was introduced by Austrian mathematician Karl Menger (1902–1985). In this conceptualization, every point in Euclidean space, denoted as E3, is assigned an integer value: 0 for a point, 1 for a straight line, 2 for a plane, and 3 for a three-dimensional figure. The second definition emanates from the work of mathematicians Felix Hausdorff (1868–1942) and Abram Besicovitch (1891–1970), who ascribed a “real number” to each natural object in E3. This real number lies between the topological dimension and the value of 3, thus providing a more nuanced characterization of the object’s complexity.

**Figure 4:**
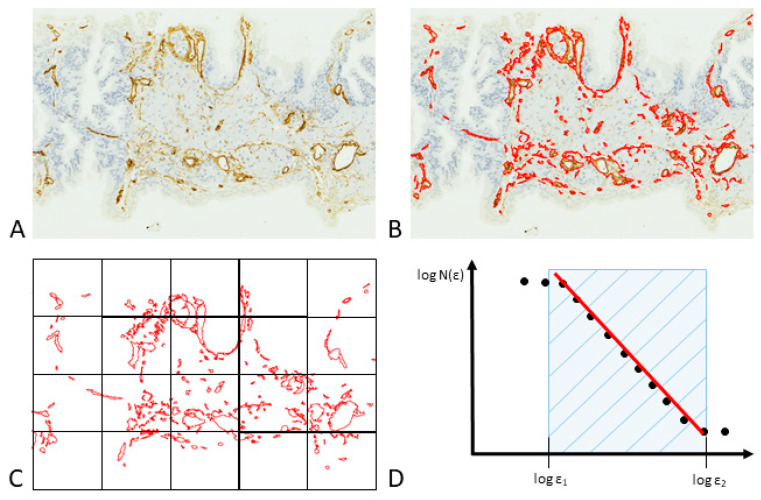
A computational method for assessing the surface fractal dimension of vascular structures in two-dimensional biopsy samples. (**A**) Sections of PCa tissue are stained with CD34-specific antibodies that selectively bind to blood vessels. (**B**) Through image segmentation, vessels that display immunoreactivity are isolated based on color similarity among adjacent pixels. (**C**) The box-counting algorithm is employed to determine the fractal dimension, denoted as Ds. This technique involves quantifying the number of square units, each with side length *ε*, necessary to fully encompass the target object, represented as *N*(*ε*). (**D**) A representative curve generated via the box-counting method delineates specific fractal windows, determined by the box sizes *ε*1 and *ε*2, which serve as the optimal interval for calculating the fractal dimension. When box sizes exceed *ε*2, they approach the image’s overall dimensions, ultimately resulting in a single box that completely covers the image. At this juncture, *N*(*ε*) becomes 1, and the slope of the curve reduces to zero. Conversely, box sizes smaller than *ε*1 approximate the image’s resolution or a single pixel; in this domain, the box-counting metric essentially reflects the image’s total area.

Mandelbrot designated the dimension of Menger’s construct using the notation D_γ_, while employing the symbol *D* for the dimensions put forth by Hausdorff and Besicovitch. In the realm of Euclidean shapes, the dimensions represented by D_γ_ and *D* are equivalent (D_γ_ = *D*). However, this equivalency does not universally extend to all fractal entities found in nature. Specifically, the inequality *D* > D_γ_ is true in such cases. Various approaches have been devised to approximate the fractal dimension of objects. Among these, the “box-counting method” (Figure 4) is most prevalently utilized in biomedical sciences as a means to estimate the spatial occupancy characteristics of anatomical structures in both two-dimensional and three-dimensional spaces [137,138,139,140,141,142,143]. It applies the following formula:

$$D=\underset{\varepsilon \to 0}{\lim}\frac{logN(\varepsilon )}{log(\frac{1}{\varepsilon })}$$

where *D* is the box-counting fractal dimension of the object, *ε* is the side length of the box, and *N*(*ε*) is the smallest number of boxes of side *ε* required to cover the outline or the surface of the object completely. Because the zero limit cannot be applied to natural objects, the dimension was estimated by the formula

*D* = d,

where d is the slope of the graph of *Log N*(*ε*) against Log (1/*ε*). The identification of linear sections within the graphs was accomplished through the utilization of regression analysis based on the least-squares method. Additionally, the slopes of these linear sections were evaluated by employing an iterative resistant-line technique. Given the intricate structure of tumor vasculature, characterized by its spatial, temporal, and molecular diversification [144], it becomes apparent that reliance solely on MVD as a metric for vascular networks is insufficient. Exploring the fundamental design principles underlying branching vascular systems, such as the human bronchial tree, can offer valuable insights for potential applications in tissue and organ engineering. This knowledge could pave the way for the development of bioartificial organs. 

In a study by Tretiakova and colleagues, the implementation of automated image analysis on both conventional and tissue microarray cross-sections in expansive, representative regions revealed no statistically significant elevations in MVD metrics when comparing PCa tissues to their corresponding normal peripheral prostatic zones [145]. Intriguingly, several morphological indices were elevated in non-cancerous prostatic glandular tissues. Further, an investigation led by Taverna et al. categorized their patient pool into two groups, one constituting 56% of the subjects exhibiting an increase in vascular surface area in PCa relative to non-tumoral regions, and another group comprising 44% of the subjects displaying a decline in vascular surface area in PCa [146]. The latter group demonstrated worse prognostic outcomes, implying that the advancement of the tumor is not necessarily contingent upon angiogenesis [146]. These observations are in alignment with recent findings by Steiner et al., which indicate no significant variances in the levels of CD31 mRNA when comparing normal prostate tissues to their cancerous counterparts (*p* = 0.78) [147]. Moreover, the absence of a meaningful correlation between mRNA and protein levels of CD31, as demonstrated through immunohistochemical analyses, suggests that angiogenic activity is relatively low in the typically slow-growing nature of PCa.

A non-intrusive imaging modality capable of illustrating MVD would offer significant advantages for both the identification and analysis of tumors [148,149]. Such an imaging technique, if it could signal a rise in MVD, would be beneficial in pinpointing optimal sites for prostate biopsies [149]. This could consequently revolutionize biopsy approaches, thereby enhancing the detection rates of PCa and facilitating more tailored treatment plans [149]. Initial findings indicate that hemodynamic metrics derived from contrast-enhanced ultrasound imaging vary between low-grade and high-grade PCa [149]. Franiel et al. endeavored to ascertain whether well-established histological markers of prognostic relevance, including MVD, exhibit a correlation with metrics acquired from pharmacokinetic dynamic contrast material-enhanced (DCE) dual-contrast-enhanced magnetic resonance (MR) imaging [150]. Their research determined that neither blood volume nor interstitial volume exhibited a consistent correlation with the histological markers, largely attributable to the uneven vascularization present in both standard prostate tissue and PCa [130]. Michallek et al. pioneered the application of fractal analysis to characterize PCa perfusion and provided evidence of its efficacy for the non-invasive prognostication of tumor grading [151]. Their study substantiated that fractal analysis applied to perfusion MRI effectively determines the grade of PCa in low-, intermediate-, and high-grade tumors, although it falls short in the highest-grade tumors. It is important to note that there is considerable variability among patients, as tumors have demonstrated both increased and diminished vascularization [152]. 

Therefore, evaluating vascularization through two-dimensional histological slides does not adequately capture the comprehensive vascular nature of the tissue [128,134]. The choice of antibody further influences the outcome, as research indicates that MVD ascertained via CD31 antibody staining yields significantly lower results than when using CD34 antibody staining [153]. Additionally, the correlation between histologic data and MR imaging is restricted by the disparity in scale; paraffin sections have a thickness of 4 μm, while the corresponding T2-weighted images possess a slice thickness of 3 mm, and the dynamic susceptibility-weighted MR DCE-MR sequence is captured with a slice thickness of 5 mm [149]. Future advancements in computer-generated 3D prostate models could potentially facilitate a more precise correlation between histologic and MR imaging data [150]. The absence of a consistent relationship between histological and functional parameters brings into question the biological relevance of metrics related to tumor microcirculation, as quantified by dynamic imaging enhanced with small-molecule contrast medium [154]. Nevertheless, Osimani et al. have recently presented evidence that blood volume and permeability surface area product measurements, obtained through perfusion computed tomography, exhibit the highest degree of correlation with immunohistochemical markers of angiogenesis in PCa. However, before these findings can be integrated into routine practice, further research involving larger datasets is required [155].

## 11. Conclusions

Almost a century ago, W.H. Lewis noted a striking divergence in both the density and morphological features of blood vessels in transplanted tumors, depending on the type of tumor in question. This led him to propose the seminal hypothesis that the vascular patterns within tumors are largely dictated by the tumor environment itself [5]. While significant advancements have been made in understanding vascular molecular and cellular biology, the physical aspects of vasculature remain relatively uncharted territory.

The vascularity of tumors in human cancers exhibits significant variability. This variation is, to some extent, indicative of the equilibrium between pro-angiogenic and anti-angiogenic factors present within the TME [156]. 

The use of MVD as a standalone predictor for angiogenesis in cancer has proven to be insufficiently effective. Recent studies employing MVD for detecting angiogenesis have yielded inconclusive results. These studies have shown a decrease in mean MVD in areas infiltrated by PCa, while areas unaffected by the cancer have shown either relatively stable or preserved angiogenic activity. 

At present, there are no explicit markers for gauging angiogenic activity in PCa, although it is plausible to consider vascular density as an indicative measure of intra-tumoral angiogenic actions. One significant limitation is the intricate geometrical architecture of the newly developed vascular network, a complexity that is challenging to interpret through two-dimensional histological slices. Potential solutions for this could include the application of fractal geometry to estimate surface dimensions, computer-aided automated image analysis, or three-dimensional modeling. It is unequivocal that the angiogenic process in PCa is more multifaceted than initially perceived. Multiple pathways are implicated, either independently or in concert, in the evolution and progression of PCa. Furthermore, PCa constitutes a complex microenvironment, consisting of diverse cell types that can have distinct, impactful roles in the neovascularization process. It is crucial to synthesize the abundant information currently accessible regarding the molecules and pathways engaged in the angiogenic process in PCa, combining it with physical concepts and the physics of cancer. Both form and function continue to serve as the foundational elements for any biological entity, influencing its alterations across spatial and temporal dimensions.

## Data Availability

Not applicable.

## References

[1] Loi S., Settleman J., Joyce J.A., Pramesh C.S., Bernards R., Fan J., Merchant J.L., Moslehi J., Sellers W.R. (2023). The next big questions in cancer research. Cell.

[2] Siegel R.L., Miller K.D., Jemal A. (2015). Cancer statistics, 2015. CA Cancer J. Clin..

[3] Salachan P.V., Rasmussen M., Ulhoi B.P., Jensen J.B., Borre M., Sorensen K.D. (2023). Spatial whole transcriptome profiling of primary tumor from patients with metastatic prostate cancer. Int. J. Cancer.

[4] Siegel R.L., Miller K.D., Fuchs H.E., Jemal A. (2022). Cancer statistics, 2022. CA Cancer J. Clin..

[5] Li P., Ferrara N. (2022). Vascular heterogeneity: VEGF receptors make blood vessels special. J. Exp. Med..

[6] Parmar D., Apte M. (2021). Angiopoietin inhibitors: A review on targeting tumor angiogenesis. Eur. J. Pharmacol..

[7] Liu Z.L., Chen H.H., Zheng L.L., Sun L.P., Shi L. (2023). Angiogenic signaling pathways and anti-angiogenic therapy for cancer. Signal Transduct. Target. Ther..

[8] Folkman J. (2007). Angiogenesis: An organizing principle for drug discovery?. Nat. Rev. Drug Discov..

[9] Ribatti D., Vacca A., Roncali L., Dammacco F. (1991). Angiogenesis under normal and pathological conditions. Haematologica.

[10] Carmeliet P. (2003). Angiogenesis in health and disease. Nat. Med..

[11] Hanahan D., Weinberg R.A. (2011). Hallmarks of cancer: The next generation. Cell.

[12] Wang W.Q., Liu L., Xu H.X., Luo G.P., Chen T., Wu C.T., Xu Y.F., Xu J., Liu C., Zhang B. (2013). Intratumoral alpha-SMA enhances the prognostic potency of cd34 associated with maintenance of microvessel integrity in hepatocellular carcinoma and pancreatic cancer. PLoS ONE.

[13] Taverna G., Grizzi F., Colombo P., Graziotti P. (2013). Is angiogenesis a hallmark of prostate cancer?. Front. Oncol..

[14] Lenzi P., Bocci G., Natale G. (2016). John Hunter and the origin of the term “angiogenesis”. Angiogenesis.

[15] Natale G., Bocci G. (2023). Discovery and development of tumor angiogenesis assays. Methods Mol. Biol..

[16] Turk J.L. (1994). Inflammation: John Hunter’s “A treatise on the blood, inflammation and gun-shot wounds”. Int. J. Exp. Pathol..

[17] Udan R.S., Culver J.C., Dickinson M.E. (2013). Understanding vascular development. Wiley Interdiscip. Rev. Dev. Biol..

[18] Fidler I.J., Ellis L.M. (2004). Neoplastic angiogenesis—not all blood vessels are created equal. N. Engl. J. Med..

[19] Benazzi C., Al-Dissi A., Chau C.H., Figg W.D., Sarli G., de Oliveira J.T., Gartner F. (2014). Angiogenesis in spontaneous tumors and implications for comparative tumor biology. Sci. World J..

[20] Karlou M., Tzelepi V., Efstathiou E. (2010). Therapeutic targeting of the prostate cancer microenvironment. Nat. Rev. Urol..

[21] Streubel B., Chott A., Huber D., Exner M., Jager U., Wagner O., Schwarzinger I. (2004). Lymphoma-specific genetic aberrations in microvascular endothelial cells in B-cell lymphomas. N. Engl. J. Med..

[22] Russo G., Mischi M., Scheepens W., De la Rosette J.J., Wijkstra H. (2012). Angiogenesis in prostate cancer: Onset, progression and imaging. BJU Int..

[23] Luczynska E., Aniol J. (2013). Neoangiogenesis in prostate cancer. Contemp. Oncol..

[24] Stifter S., Patrinicola F., Taverna G., Grizzi F. (2017). Angiogenesis and Prostate Cancer: Friends or Foes. Biochemical Basis and Therapeutic Implications of Angiogenesis.

[25] Melegh Z., Oltean S. (2019). Targeting angiogenesis in prostate cancer. Int. J. Mol. Sci..

[26] Gong Y., Chippada-Venkata U.D., Oh W.K. (2014). Roles of matrix metalloproteinases and their natural inhibitors in prostate cancer progression. Cancers.

[27] Wang M., Wisniewski C.A., Xiong C., Chhoy P., Goel H.L., Kumar A., Zhu L.J., Li R., St Louis P.A., Ferreira L.M. (2023). Therapeutic blocking of VEGF binding to neuropilin-2 diminishes PD-L1 expression to activate antitumor immunity in prostate cancer. Sci. Transl. Med..

[28] Ryan C.J., Dutta S., Kelly W.K., Middleberg R., Russell C., Morris M.J., Taplin M.E., Halabi S., Alliance for Clinical Trials in Oncology Genitourinary Committee (2020). Androgens and overall survival in patients with metastatic castration-resistant prostate cancer treated with docetaxel. Clin. Genitourin. Cancer.

[29] Sarkar C., Goswami S., Basu S., Chakroborty D. (2020). Angiogenesis inhibition in prostate cancer: An update. Cancers.

[30] Zhang J., Lu T., Lu S., Ma S., Han D., Zhang K., Xu C., Liu S., Gan L., Wu X. (2023). Single-cell analysis of multiple cancer types reveals differences in endothelial cells between tumors and normal tissues. Comput. Struct. Biotechnol. J..

[31] Sorrentino C., Di Carlo E. (2023). Molecular targeted therapies in metastatic prostate cancer: Recent advances and future challenges. Cancers.

[32] Jamaspishvili T., Kral M., Khomeriki I., Student V., Kolar Z., Bouchal J. (2009). Urine markers in monitoring for prostate cancer. Prostate Cancer Prostatic Dis..

[33] George D.J., Halabi S., Shepard T.F., Vogelzang N.J., Hayes D.F., Small E.J., Kantoff P.W., Cancer and B. (2001). Leukemia Group. Prognostic significance of plasma vascular endothelial growth factor levels in patients with hormone-refractory prostate cancer treated on Cancer and Leukemia Group B 9480. Clin. Cancer Res..

[34] Jariani A., Kakroodi S.T., Arabfard M., Jamialahmadi T., Rahimi M., Sahebkar A. (2023). Identification of key genes in angiogenesis of breast and prostate cancers in the context of different cell types. Curr. Med. Chem..

[35] Yang Y., Li J., Lei W., Wang H., Ni Y., Liu Y., Yan H., Tian Y., Wang Z., Yang Z. (2023). CXCL12-CXCR4/CXCR7 axis in cancer: From mechanisms to clinical applications. Int. J. Biol. Sci..

[36] Heidegger I., Fotakis G., Offermann A., Goveia J., Daum S., Salcher S., Noureen A., Timmer-Bosscha H., Schafer G., Walenkamp A. (2022). Comprehensive characterization of the prostate tumor microenvironment identifies CXCR4/CXCL12 crosstalk as a novel antiangiogenic therapeutic target in prostate cancer. Mol. Cancer.

[37] Uygur B., Wu W.S. (2011). SLUG promotes prostate cancer cell migration and invasion via CXCR4/CXCL12 axis. Mol. Cancer.

[38] Dome B., Hendrix M.J., Paku S., Tovari J., Timar J. (2007). Alternative vascularization mechanisms in cancer: Pathology and therapeutic implications. Am. J. Pathol..

[39] Carmeliet P., Jain R.K. (2011). Molecular mechanisms and clinical applications of angiogenesis. Nature.

[40] Dudley A.C., Griffioen A.W. (2023). Pathological angiogenesis: Mechanisms and therapeutic strategies. Angiogenesis.

[41] Luo F., Yang K., Liu R.L., Meng C., Dang R.F., Xu Y. (2014). Formation of vasculogenic mimicry in bone metastasis of prostate cancer: Correlation with cell apoptosis and senescence regulation pathways. Pathol. Res. Pract..

[42] Cuypers A., Truong A.K., Becker L.M., Saavedra-Garcia P., Carmeliet P. (2022). Tumor vessel co-option: The past & the future. Front. Oncol..

[43] Ausprunk D.H., Folkman J. (1977). Migration and proliferation of endothelial cells in preformed and newly formed blood vessels during tumor angiogenesis. Microvasc. Res..

[44] Germain S., Monnot C., Muller L., Eichmann A. (2010). Hypoxia-driven angiogenesis: Role of tip cells and extracellular matrix scaffolding. Curr. Opin. Hematol..

[45] Terrassoux L., Claux H., Bacari S., Meignan S., Furlan A. (2022). A Bloody conspiracy- blood vessels and immune cells in the tumor microenvironment. Cancers.

[46] Sokoloff M.H., Chung L.W. (1998). Targeting angiogenic pathways involving tumor-stromal interaction to treat advanced human prostate cancer. Cancer Metastasis Rev..

[47] Kryza T., Parent C., Pardessus J., Petit A., Burlaud-Gaillard J., Reverdiau P., Iochmann S., Labas V., Courty Y., Heuze-Vourc’h N. (2018). Human kallikrein-related peptidase 12 stimulates endothelial cell migration by remodeling the fibronectin matrix. Sci. Rep..

[48] Brennen W.N., Nguyen H., Dalrymple S.L., Reppert-Gerber S., Kim J., Isaacs J.T., Hammers H. (2016). Assessing angiogenic responses induced by primary human prostate stromal cells in a three-dimensional fibrin matrix assay. Oncotarget.

[49] Mukai H., Muramatsu A., Mashud R., Kubouchi K., Tsujimoto S., Hongu T., Kanaho Y., Tsubaki M., Nishida S., Shioi G. (2016). PKN3 is the major regulator of angiogenesis and tumor metastasis in mice. Sci. Rep..

[50] Djonov V., Baum O., Burri P.H. (2003). Vascular remodeling by intussusceptive angiogenesis. Cell Tissue Res..

[51] Lugano R., Ramachandran M., Dimberg A. (2020). Tumor angiogenesis: Causes, consequences, challenges and opportunities. Cell Mol. Life Sci..

[52] Kurz H., Burri P.H., Djonov V.G. (2003). Angiogenesis and vascular remodeling by intussusception: From form to function. News Physiol. Sci..

[53] Djonov V., Schmid M., Tschanz S.A., Burri P.H. (2000). Intussusceptive angiogenesis: Its role in embryonic vascular network formation. Circ. Res..

[54] Ribatti D., Djonov V. (2012). Intussusceptive microvascular growth in tumors. Cancer Lett..

[55] Osawa M., Masuda M., Kusano K., Fujiwara K. (2002). Evidence for a role of platelet endothelial cell adhesion molecule-1 in endothelial cell mechanosignal transduction: Is it a mechanoresponsive molecule?. J. Cell Biol..

[56] Diaz-Flores L., Gutierrez R., Garcia M.P., Gonzalez-Gomez M., Diaz-Flores L., Carrasco J.L., Madrid J.F., Rodriguez Bello A. (2022). Comparison of the behavior of perivascular cells (pericytes and cd34+ stromal cell/telocytes) in sprouting and intussusceptive angiogenesis. Int. J. Mol. Sci..

[57] Gueron G., Cotignola J., Vazquez E. (2013). Inflammatory Microenvironment in Prostate Carcinogenesis. Advances in Prostate Cancer.

[58] Nico B., Crivellato E., Guidolin D., Annese T., Longo V., Finato N., Vacca A., Ribatti D. (2010). Intussusceptive microvascular growth in human glioma. Clin. Exp. Med..

[59] Ribatti D., Nico B., Floris C., Mangieri D., Piras F., Ennas M.G., Vacca A., Sirigu P. (2005). Microvascular density, vascular endothelial growth factor immunoreactivity in tumor cells, vessel diameter and intussusceptive microvascular growth in primary melanoma. Oncol. Rep..

[60] Patan S., Munn L.L., Jain R.K. (1996). Intussusceptive microvascular growth in a human colon adenocarcinoma xenograft: A novel mechanism of tumor angiogenesis. Microvasc. Res..

[61] Patan S. (2004). Vasculogenesis and angiogenesis. Cancer Treat. Res..

[62] Patan S. (2000). Vasculogenesis and angiogenesis as mechanisms of vascular network formation, growth and remodeling. J. Neurooncol..

[63] Kolte D., McClung J.A., Aronow W.S. (2016). Vasculogenesis and Angiogenesis. Translational Research in Coronary Artery Disease.

[64] Wang K., Peng H.L., Li L.K. (2012). Prognostic value of vascular endothelial growth factor expression in patients with prostate cancer: A systematic review with meta-analysis. Asian Pac. J. Cancer Prev..

[65] Yang L., You S., Kumar V., Zhang C., Cao Y. (2012). In vitro the behaviors of metastasis with suppression of VEGF in human bone metastatic LNCaP-derivative C4-2B prostate cancer cell line. J. Exp. Clin. Cancer Res..

[66] Huang S., Peng L., Tang Y., Zhang L., Guo W., Zou X., Peng X. (2013). Hypoxia of PC-3 prostate cancer cells enhances migration and vasculogenesis in vitro of bone marrow-derived endothelial progenitor cells by secretion of cytokines. Oncol. Rep..

[67] Huang S., He P., Peng X., Li J., Xu D., Tang Y. (2015). Pristimerin inhibits prostate cancer bone metastasis by targeting PC-3 stem cell characteristics and VEGF-induced vasculogenesis of BM-EPCs. Cell Physiol. Biochem..

[68] Huang S., Tang Y., Peng X., Cai X., Wa Q., Ren D., Li Q., Luo J., Li L., Zou X. (2016). Acidic extracellular pH promotes prostate cancer bone metastasis by enhancing PC-3 stem cell characteristics, cell invasiveness and VEGF-induced vasculogenesis of BM-EPCs. Oncol. Rep..

[69] Chen F.-H., Chiang C.-S., Wang C.-C., Tsai C.-S., Jung S.-M., Lee C.-C., McBride W.H., Hong J.-H. (2009). Radiotherapy decreases vascular density and causes hypoxia with macrophage aggregation in TRAMP-C1 prostate tumors. Clin. Cancer Res..

[70] Pezzella F., Di Bacco A., Andreola S., Nicholson A.G., Pastorino U., Harris A.L. (1996). Angiogenesis in primary lung cancer and lung secondaries. Eur. J. Cancer.

[71] Chang Y.S., di Tomaso E., McDonald D.M., Jones R., Jain R.K., Munn L.L. (2000). Mosaic blood vessels in tumors: Frequency of cancer cells in contact with flowing blood. Proc. Natl. Acad. Sci. USA.

[72] Plate K.H., Scholz A., Dumont D.J. (2012). Tumor angiogenesis and anti-angiogenic therapy in malignant gliomas revisited. Acta Neuropathol..

[73] Thompson W.D., Shiach K.J., Fraser R.A., McIntosh L.C., Simpson J.G. (1987). Tumours acquire their vasculature by vessel incorporation, not vessel ingrowth. J. Pathol..

[74] Holash J., Maisonpierre P.C., Compton D., Boland P., Alexander C.R., Zagzag D., Yancopoulos G.D., Wiegand S.J. (1999). Vessel cooption, regression, and growth in tumors mediated by angiopoietins and VEGF. Science.

[75] Pezzella F., Pastorino U., Tagliabue E., Andreola S., Sozzi G., Gasparini G., Menard S., Gatter K.C., Harris A.L., Fox S. (1997). Non-small-cell lung carcinoma tumor growth without morphological evidence of neo-angiogenesis. Am. J. Pathol..

[76] Dome B., Paku S., Somlai B., Timar J. (2002). Vascularization of cutaneous melanoma involves vessel co-option and has clinical significance. J. Pathol..

[77] Lugassy C., Vernon S.E., Busam K., Engbring J.A., Welch D.R., Poulos E.G., Kleinman H.K., Barnhill R.L. (2006). Angiotropism of human melanoma: Studies involving in transit and other cutaneous metastases and the chicken chorioallantoic membrane: Implications for extravascular melanoma invasion and metastasis. Am. J. Dermatopathol..

[78] Cao Z., Bao M., Miele L., Sarkar F.H., Wang Z., Zhou Q. (2013). Tumour vasculogenic mimicry is associated with poor prognosis of human cancer patients: A systemic review and meta-analysis. Eur. J. Cancer.

[79] Maniotis A.J., Folberg R., Hess A., Seftor E.A., Gardner L.M., Pe’er J., Trent J.M., Meltzer P.S., Hendrix M.J. (1999). Vascular channel formation by human melanoma cells in vivo and in vitro: Vasculogenic mimicry. Am. J. Pathol..

[80] Seftor R.E., Hess A.R., Seftor E.A., Kirschmann D.A., Hardy K.M., Margaryan N.V., Hendrix M.J. (2012). Tumor cell vasculogenic mimicry: From controversy to therapeutic promise. Am. J. Pathol..

[81] Folberg R., Maniotis A.J. (2004). Vasculogenic mimicry. APMIS.

[82] Zhang S., Zhang D., Sun B. (2007). Vasculogenic mimicry: Current status and future prospects. Cancer Lett..

[83] Liu R., Yang K., Meng C., Zhang Z., Xu Y. (2012). Vasculogenic mimicry is a marker of poor prognosis in prostate cancer. Cancer Biol. Ther..

[84] Sharma N., Seftor R.E., Seftor E.A., Gruman L.M., Heidger P.M., Cohen M.B., Lubaroff D.M., Hendrix M.J. (2002). Prostatic tumor cell plasticity involves cooperative interactions of distinct phenotypic subpopulations: Role in vasculogenic mimicry. Prostate.

[85] Yang C., Jiang L., Zhang H., Shimoda L.A., DeBerardinis R.J., Semenza G.L. (2014). Analysis of hypoxia-induced metabolic reprogramming. Methods Enzymol..

[86] Han D.S., Lee H.J., Lee E.O. (2022). Resveratrol suppresses serum-induced vasculogenic mimicry through impairing the EphA2/twist-VE-cadherin/AKT pathway in human prostate cancer PC-3 cells. Sci. Rep..

[87] Wang H., Lin H., Pan J., Mo C., Zhang F., Huang B., Wang Z., Chen X., Zhuang J., Wang D. (2016). Vasculogenic mimicry in prostate cancer: The roles of EphA2 and PI3K. J. Cancer.

[88] Ahmadi S.A., Moinfar M., Gohari Moghaddam K., Bahadori M. (2010). Practical application of angiogenesis and vasculogenic mimicry in prostatic adenocarcinoma. Arch. Iran. Med..

[89] Han D.-S., Lee E.-O. (2022). Sp1 Plays a key role in vasculogenic mimicry of human prostate cancer cells. Int. J. Mol. Sci..

[90] Potenta S., Zeisberg E., Kalluri R. (2008). The role of endothelial-to-mesenchymal transition in cancer progression. Br. J. Cancer.

[91] Kalluri R., Zeisberg M. (2006). Fibroblasts in cancer. Nat. Rev. Cancer.

[92] Bonollo F., Thalmann G.N., Kruithof-de Julio M., Karkampouna S. (2020). The role of cancer-associated fibroblasts in prostate cancer tumorigenesis. Cancers.

[93] Olumi A.F., Grossfeld G.D., Hayward S.W., Carroll P.R., Tlsty T.D., Cunha G.R. (1999). Carcinoma-associated fibroblasts direct tumor progression of initiated human prostatic epithelium. Cancer Res..

[94] Thalmann G.N., Rhee H., Sikes R.A., Pathak S., Multani A., Zhau H.E., Marshall F.F., Chung L.W. (2010). Human prostate fibroblasts induce growth and confer castration resistance and metastatic potential in LNCaP Cells. Eur. Urol..

[95] Turpin A., Duterque-Coquillaud M., Vieillard M.H. (2020). Bone metastasis: Current state of play. Transl. Oncol..

[96] Conteduca V., Oromendia C., Eng K.W., Bareja R., Sigouros M., Molina A., Faltas B.M., Sboner A., Mosquera J.M., Elemento O. (2019). Clinical features of neuroendocrine prostate cancer. Eur. J. Cancer.

[97] Bedeschi M., Marino N., Cavassi E., Piccinini F., Tesei A. (2023). Cancer-associated fibroblast: Role in prostate cancer progression to metastatic disease and therapeutic resistance. Cells.

[98] Chang Z., Cui J., Song Y. (2018). Long noncoding RNA PVT1 promotes EMT via mediating microRNA-186 targeting of Twist1 in prostate cancer. Gene.

[99] Apeldoorn C., Safaei S., Paton J., Maso Talou G.D. (2022). Computational models for generating microvascular structures: Investigations beyond medical imaging resolution. WIREs Mech. Dis..

[100] Korn C., Augustin H.G. (2015). Mechanisms of Vessel Pruning and Regression. Dev. Cell.

[101] Franco C.A., Jones M.L., Bernabeu M.O., Geudens I., Mathivet T., Rosa A., Lopes F.M., Lima A.P., Ragab A., Collins R.T. (2015). Dynamic endothelial cell rearrangements drive developmental vessel regression. PLoS Biol..

[102] Bergers G., Hanahan D. (2008). Modes of resistance to anti-angiogenic therapy. Nat. Rev. Cancer.

[103] Hirota K., Semenza G.L. (2006). Regulation of angiogenesis by hypoxia-inducible factor 1. Crit. Rev. Oncol. Hematol..

[104] Potiron V.A., Abderrahmani R., Clement-Colmou K., Marionneau-Lambot S., Oullier T., Paris F., Supiot S. (2013). Improved functionality of the vasculature during conventionally fractionated radiation therapy of prostate cancer. PLoS ONE.

[105] Imaizumi N., Monnier Y., Hegi M., Mirimanoff R.O., Ruegg C. (2010). Radiotherapy suppresses angiogenesis in mice through TGF-betaRI/ALK5-dependent inhibition of endothelial cell sprouting. PLoS ONE.

[106] El-Kenawi A.E., El-Remessy A.B. (2013). Angiogenesis inhibitors in cancer therapy: Mechanistic perspective on classification and treatment rationales. Br. J. Pharmacol..

[107] Mundel T.M., Kalluri R. (2007). Type IV collagen-derived angiogenesis inhibitors. Microvasc. Res..

[108] Minder P., Zajac E., Quigley J.P., Deryugina E.I. (2015). EGFR regulates the development and microarchitecture of intratumoral angiogenic vasculature capable of sustaining cancer cell intravasation. Neoplasia.

[109] Krebs M., Solimando A.G., Kalogirou C., Marquardt A., Frank T., Sokolakis I., Hatzichristodoulou G., Kneitz S., Bargou R., Kubler H. (2020). miR-221-3p regulates VEGFR2 expression in high-risk prostate cancer and represents an escape mechanism from sunitinib in vitro. J. Clin. Med..

[110] Huss W.J., Hanrahan C.F., Barrios R.J., Simons J.W., Greenberg N.M. (2001). Angiogenesis and prostate cancer: Identification of a molecular progression switch. Cancer Res..

[111] Annese T., Tamma R., De Giorgis M., Ribatti D. (2020). microRNAs biogenesis, functions and role in tumor angiogenesis. Front. Oncol..

[112] Folkman J. (1971). Tumor angiogenesis: Therapeutic implications. N. Engl. J. Med..

[113] Folkman J. (1990). What is the evidence that tumors are angiogenesis dependent?. J. Natl. Cancer Inst..

[114] Weidner N., Semple J.P., Welch W.R., Folkman J. (1991). Tumor angiogenesis and metastasis—correlation in invasive breast carcinoma. N. Engl. J. Med..

[115] Jones T.A., Radtke J.P., Hadaschik B., Marks L.S. (2016). Optimizing safety and accuracy of prostate biopsy. Curr. Opin. Urol..

[116] Jiang J., Li J., Xiong X., Zhang S., Tan D., Yang L., Wei Q. (2023). Different predictive values of microvessel density for biochemical recurrence among different PCa populations: A systematic review and meta-analysis. Cancer Med..

[117] Wang B., Pan D., Ban Y., Sun Z., Tian Y., Luo G. (2022). The relationship between prostatic microvessel density and different concentrations of oestrogen/androgen in Sprague-Dawley rats. Eur. J. Med. Res..

[118] Miyata Y., Sakai H. (2015). Reconsideration of the clinical and histopathological significance of angiogenesis in prostate cancer: Usefulness and limitations of microvessel density measurement. Int. J. Urol..

[119] Grivas N., Goussia A., Stefanou D., Giannakis D. (2016). Microvascular density and immunohistochemical expression of VEGF, VEGFR-1 and VEGFR-2 in benign prostatic hyperplasia, high-grade prostate intraepithelial neoplasia and prostate cancer. Cent. Eur. J. Urol..

[120] Taverna G., Cote R.J., Grizzi F. (2015). Editorial: Prostate cancer: What we know and what we would like to know. Front. Oncol..

[121] McCullough D.J., Stabley J.N., Siemann D.W., Behnke B.J. (2014). Modulation of blood flow, hypoxia, and vascular function in orthotopic prostate tumors during exercise. J. Natl. Cancer Inst..

[122] Djurhuus S.S., Schauer T., Simonsen C., Toft B.G., Jensen A.R.D., Erler J.T., Røder M.A., Hojman P., Brasso K., Christensen J.F. (2022). Effects of acute exercise training on tumor outcomes in men with localized prostate cancer: A randomized controlled trial. Physiol. Rep..

[123] Das B., Mendonca S.M. (2022). Prognostic significance of mast cells and vascular density in prostatic adenocarcinoma. Indian J. Pathol. Microbiol..

[124] Hlatky L., Hahnfeldt P., Folkman J. (2002). Clinical application of antiangiogenic therapy: Microvessel density, what it does and doesn’t tell us. J. Natl. Cancer Inst..

[125] Eberhard A., Kahlert S., Goede V., Hemmerlein B., Plate K.H., Augustin H.G. (2000). Heterogeneity of angiogenesis and blood vessel maturation in human tumors: Implications for antiangiogenic tumor therapies. Cancer Res..

[126] Erbersdobler A., Isbarn H., Dix K., Steiner I., Schlomm T., Mirlacher M., Sauter G., Haese A. (2010). Prognostic value of microvessel density in prostate cancer: A tissue microarray study. World J. Urol..

[127] Preusser M., Heinzl H., Gelpi E., Schonegger K., Haberler C., Birner P., Marosi C., Hegi M., Gorlia T., Hainfellner J.A. (2006). Histopathologic assessment of hot-spot microvessel density and vascular patterns in glioblastoma: Poor observer agreement limits clinical utility as prognostic factors: A translational research project of the european organization for research and treatment of cancer brain tumor group. Cancer.

[128] Rubin M.A., Buyyounouski M., Bagiella E., Sharir S., Neugut A., Benson M., de la Taille A., Katz A.E., Olsson C.A., Ennis R.D. (1999). Microvessel density in prostate cancer: Lack of correlation with tumor grade, pathologic stage, and clinical outcome. Urology.

[129] Pluda J.M. (1997). Tumor-associated angiogenesis: Mechanisms, clinical implications, and therapeutic strategies. Semin. Oncol..

[130] Mucci L.A., Powolny A., Giovannucci E., Liao Z., Kenfield S.A., Shen R., Stampfer M.J., Clinton S.K. (2009). Prospective study of prostate tumor angiogenesis and cancer-specific mortality in the health professionals follow-up study. J. Clin. Oncol..

[131] Aird W.C. (2012). Endothelial cell heterogeneity. Cold Spring Harb. Perspect. Med..

[132] Grizzi F., Colombo P., Taverna G., Chiriva-Internati M., Cobos E., Graziotti P., Muzzio P.C., Dioguardi N. (2007). Geometry of human vascular system: Is it an obstacle for quantifying antiangiogenic therapies?. Appl. Immunohistochem. Mol. Morphol..

[133] Baish J.W., Jain R.K. (2000). Fractals and cancer. Cancer Res..

[134] Grizzi F., Russo C., Colombo P., Franceschini B., Frezza E.E., Cobos E., Chiriva-Internati M. (2005). Quantitative evaluation and modeling of two-dimensional neovascular network complexity: The surface fractal dimension. BMC Cancer.

[135] Grizzi F., Spadaccini M., Chiriva-Internati M., Hegazi M., Bresalier R.S., Hassan C., Repici A., Carrara S. (2023). Fractal nature of human gastrointestinal system: Exploring a new era. World J. Gastroenterol..

[136] Bassingthwaighte J.B. (1992). Fractal Vascular Growth Patterns. Acta Ster..

[137] Losa G.A., Graber R., Baumann G., Nonnenmacher T.F. (1998). Steroid hormones modify nuclear heterochromatin structure and plasma membrane enzyme of MCF-7 cells. A combined fractal, electron microscopical and enzymatic analysis. Eur. J. Histochem..

[138] Losa G.A. (2002). Fractal morphometry of cell complexity. Riv. Biol..

[139] Losa G.A., Nonnenmacher T.F. (1996). Self-similarity and fractal irregularity in pathologic tissues. Mod. Pathol..

[140] Grizzi F., Fiorino S., Qehajaj D., Fornelli A., Russo C., de Biase D., Masetti M., Mastrangelo L., Zanello M., Lombardi R. (2019). Computer-aided assessment of the extra-cellular matrix during pancreatic carcinogenesis: A pilot study. J. Transl. Med..

[141] Di Ieva A., Grizzi F., Sherif C., Matula C., Tschabitscher M. (2011). Angioarchitectural heterogeneity in human glioblastoma multiforme: A fractal-based histopathological assessment. Microvasc. Res..

[142] Dioguardi N., Grizzi F., Bossi P., Roncalli M. (1999). Fractal and spectral dimension analysis of liver fibrosis in needle biopsy specimens. Anal. Quant. Cytol. Histol..

[143] Cross S.S. (1997). Fractals in pathology. J. Pathol..

[144] Perez-Gutierrez L., Li P., Ferrara N. (2022). Endothelial cell diversity: The many facets of the crystal. FEBS J..

[145] Tretiakova M., Antic T., Binder D., Kocherginsky M., Liao C., Taxy J.B., Oto A. (2013). Microvessel density is not increased in prostate cancer: Digital imaging of routine sections and tissue microarrays. Hum. Pathol..

[146] Taverna G., Colombo P., Grizzi F., Franceschini B., Ceva-Grimaldi G., Seveso M., Giusti G., Piccinelli A., Graziotti P. (2009). Fractal analysis of two-dimensional vascularity in primary prostate cancer and surrounding non-tumoral parenchyma. Pathol. Res. Pract..

[147] Steiner I., Jung K., Miller K., Stephan C., Erbersdobler A. (2012). Expression of endothelial factors in prostate cancer: A possible role of caveolin-1 for tumour progression. Oncol. Rep..

[148] Jain R.K. (1997). The Eugene M. Landis Award Lecture 1996. Delivery of molecular and cellular medicine to solid tumors. Microcirculation.

[149] Jiang J., Chen Y., Zhu Y., Yao X., Qi J. (2011). Contrast-enhanced ultrasonography for the detection and characterization of prostate cancer: Correlation with microvessel density and Gleason score. Clin. Radiol..

[150] Franiel T., Ludemann L., Rudolph B., Rehbein H., Stephan C., Taupitz M., Beyersdorff D. (2009). Prostate MR imaging: Tissue characterization with pharmacokinetic volume and blood flow parameters and correlation with histologic parameters. Radiology.

[151] Michallek F., Huisman H., Hamm B., Elezkurtaj S., Maxeiner A., Dewey M. (2022). Prediction of prostate cancer grade using fractal analysis of perfusion MRI: Retrospective proof-of-principle study. Eur. Radiol..

[152] van der Kwast T.H., Roobol M.J. (2015). Prostate cancer: Is prostatectomy for Gleason score 6 a treatment failure?. Nat. Rev. Urol..

[153] de la Taille A., Katz A.E., Bagiella E., Buttyan R., Sharir S., Olsson C.A., Burchardt T., Ennis R.D., Rubin M.A. (2000). Microvessel density as a predictor of PSA recurrence after radical prostatectomy. A comparison of CD34 and CD31. Am. J. Clin. Pathol..

[154] Cyran C.C., von Einem J.C., Paprottka P.M., Schwarz B., Ingrisch M., Dietrich O., Hinkel R., Bruns C.J., Clevert D.A., Eschbach R. (2012). Dynamic contrast-enhanced computed tomography imaging biomarkers correlated with immunohistochemistry for monitoring the effects of sorafenib on experimental prostate carcinomas. Invest. Radiol..

[155] Osimani M., Bellini D., Di Cristofano C., Palleschi G., Petrozza V., Carbone A., Laghi A. (2012). Perfusion MDCT of prostate cancer: Correlation of perfusion CT parameters and immunohistochemical markers of angiogenesis. AJR Am. J. Roentgenol..

[156] Gupta J., Tayyib N.A., Jalil A.T., Hlail S.H., Zabibah R.S., Vokhidov U.N., Alsaikhan F., Ramaiah P., Chinnasamy L., Kadhim M.M. (2023). Angiogenesis and prostate cancer: MicroRNAs comes into view. Pathol. Res. Pract..

